# Delays and loss to follow-up before treatment of drug-resistant tuberculosis following implementation of Xpert MTB/RIF in South Africa: A retrospective cohort study

**DOI:** 10.1371/journal.pmed.1002238

**Published:** 2017-02-21

**Authors:** Helen Cox, Lindy Dickson-Hall, Norbert Ndjeka, Anja van’t Hoog, Alison Grant, Frank Cobelens, Wendy Stevens, Mark Nicol

**Affiliations:** 1 Division of Medical Microbiology, Department of Pathology, University of Cape Town, Cape Town, South Africa; 2 Institute of Infectious Disease and Molecular Medicine, University of Cape Town, Cape Town, South Africa; 3 National Department of Health, Pretoria, South Africa; 4 Amsterdam Institute for Global Health and Development, Amsterdam, The Netherlands; 5 Department of Global Health, Academic Medical Centre, University of Amsterdam, Amsterdam, The Netherlands; 6 Department of Clinical Research, London School of Hygiene & Tropical Medicine, London, United Kingdom; 7 School of Nursing & Public Health, Africa Health Research Institute, University of KwaZulu-Natal, Durban, South Africa; 8 School of Public Health, Faculty of Health Sciences, University of the Witwatersrand, Johannesburg, South Africa; 9 National Priority Programmes Unit, National Health Laboratory Service, Johannesburg, South Africa; 10 National Health Laboratory Service, Cape Town, South Africa; Centers for Disease Control and Prevention, UNITED STATES

## Abstract

**Background:**

South Africa has a large burden of rifampicin-resistant tuberculosis (RR-TB), with 18,734 patients diagnosed in 2014. The number of diagnosed patients has increased substantially with the introduction of the Xpert MTB/RIF test, used for tuberculosis (TB) diagnosis for all patients with presumptive TB. Routine aggregate data suggest a large treatment gap (pre-treatment loss to follow-up) between the numbers of patients with laboratory-confirmed RR-TB and those reported to have started second-line treatment. We aimed to assess the impact of Xpert MTB/RIF implementation on the delay to treatment initiation and loss to follow-up before second-line treatment for RR-TB across South Africa.

**Methods and findings:**

A nationwide retrospective cohort study was conducted to assess second-line treatment initiation and treatment delay among laboratory-diagnosed RR-TB patients. Cohorts, including approximately 300 sequentially diagnosed RR-TB patients per South African province, were drawn from the years 2011 and 2013, i.e., before and after Xpert implementation. Patients with prior laboratory RR-TB diagnoses within 6 mo and currently treated patients were excluded. Treatment initiation was determined through data linkage with national and local treatment registers, medical record review, interviews with health care staff, and direct contact with patients or household members. Additional laboratory data were used to track cases. National estimates of the percentage of patients who initiated treatment and time to treatment were weighted to account for the sampling design.

There were 2,508 and 2,528 eligible patients in the 2011 and 2013 cohorts, respectively; 92% were newly diagnosed with RR-TB (no prior RR-TB diagnoses). Nationally, among the 2,340 and 2,311 new RR-TB patients in the 2011 and 2013 cohorts, 55% (95% CI 53%–57%) and 63% (95% CI 61%–65%), respectively, started treatment within 6 mo of laboratory receipt of their diagnostic specimen (*p <* 0.001). However, in 2013, there was no difference in the percentage of patients who initiated treatment at 6 mo between the 1,368 new RR-TB patients diagnosed by Xpert (62%, 95% CI 59%–65%) and the 943 diagnosed by other methods (64%, 95% CI 61%–67%) (*p* = 0.39). The median time to treatment decreased from 44 d (interquartile range [IQR] 20–69) in 2011 to 22 d (IQR 2–43) in 2013 (*p <* 0.001). In 2013, across the nine provinces, there were substantial variations in both treatment initiation (range 51%–73% by 6 mo) and median time to treatment (range 15–36 d, *n* = 1,450), and only 53% of the 1,448 new RR-TB patients who received treatment were recorded in the national RR-TB register.

This retrospective study is limited by the lack of information to assess reasons for non-initiation of treatment, particularly pre-treatment mortality data. Other limitations include the use of names and dates of birth to locate patient-level data, potentially resulting in missed treatment initiation among some patients.

**Conclusions:**

In 2013, there was a large treatment gap for RR-TB in South Africa that varied significantly across provinces. Xpert implementation, while reducing treatment delay, had not contributed substantially to reducing the treatment gap in 2013. However, given improved case detection with Xpert, a larger proportion of RR-TB patients overall have received treatment, with reduced delays. Nonetheless, strategies to further improve linkage to treatment for all diagnosed RR-TB patients are urgently required.

## Abstract 2

Helen Cox and colleagues investigate whether the treatment gap for rifampicin-resistant tuberculosis has changed following implementation of Xpert MTB/RIF in South Africa.

## Author Summary

**Why was this study done?:**

- Tuberculosis (TB) that is resistant to rifampicin, one of the most important first-line TB drugs, is more difficult to treat than drug-susceptible TB and requires treatment with second-line TB drugs. South Africa diagnoses close to 20,000 patients with rifampicin-resistant TB each year, but routine data suggest that a large proportion of these patients do not receive adequate treatment (treatment gap).
- South Africa has implemented the Xpert MTB/RIF test (a rapid test for TB and rifampicin resistance) for all individuals being investigated for TB. Prior to this, only a subset of TB patients received drug susceptibility testing, and the diagnosis of drug-resistant TB was often delayed, resulting in delayed treatment initiation.
- In South Africa, the number of diagnosed rifampicin-resistant TB patients is derived from laboratory data, while the number of patients who receive second-line treatment is derived from a separate treatment register. As both of these data sources provide aggregate numbers that are not linked, we do not have an accurate estimate of the proportion of patients who receive second-line treatment.
- Use of Xpert was expected to both increase case detection for rifampicin-resistant TB and reduce the delay to second-line treatment initiation, thereby increasing the proportion of diagnosed patients who receive treatment.

**What did the researchers do and find?:**

- A retrospective cohort study was conducted to quantify the proportion of diagnosed patients who received second-line treatment and the delay to treatment.
- Two cohorts of approximately 2,500 patients each were identified: the first in 2011 prior to Xpert implementation and the second in 2013 after Xpert implementation.
- While treatment initiation increased from 55% in 2011 to 63% in 2013, the percentage of patients starting second-line treatment was low overall and varied considerably across the nine provinces of South Africa.
- Xpert contributed to a reduction in treatment delay from a median of 44 days in 2011 to 22 days in 2013, but did not significantly improve the proportion receiving treatment.

**What do these findings mean?:**

- Use of Xpert contributes to reduced delays to treatment initiation but does not reduce the treatment gap. However, given improved case detection, a larger proportion of the total population of individuals with rifampicin-resistant TB will receive treatment.
- Further strategies to improve linkage to treatment for all patients diagnosed with rifampicin-resistant TB are required.

## Introduction

The global epidemic of drug-resistant tuberculosis (DR-TB) is largely undiagnosed and untreated. In 2014, barely 23% of the estimated incident cases of multidrug-resistant tuberculosis or rifampicin-resistant tuberculosis (RR-TB) were initiated on appropriate second-line anti-tuberculosis therapy [1]. The remainder contribute to ongoing transmission [2] and suffer high mortality. At a global level, the major bottleneck in the cascade from diagnosis through to cure is access to drug susceptibility testing (DST); only 12% of previously untreated tuberculosis (TB) patients are reported to receive any DST [1]. Subsequent gaps in the cascade occur due to poor access to treatment and poor outcomes with currently available second-line treatment [1].

The rollout of the Xpert MTB/RIF test has increased access to DST in several high-burden settings, including South Africa. Xpert provides rapid diagnosis of RR-TB, leading to results being available to clinicians in days rather than weeks or months, as with culture-based methods [3]. Earlier diagnosis should allow earlier initiation of second-line treatment and potentially reduce the proportion of patients who never start treatment (pre-treatment loss to follow-up), potentially improving patient outcomes and reducing the risk of transmission [4,5]. Diagnosing a greater proportion of the estimated community burden of RR-TB, initiating second-line treatment for a larger proportion of those who are diagnosed, and subsequently providing successful treatment are all important elements for improving the DR-TB care cascade.

South Africa has a large burden of RR-TB; in 2014, 18,734 RR-TB cases were notified to the World Health Organization (WHO), considerably more than the number of incident RR-TB cases estimated by WHO to occur [1]. This represents a substantial increase over previous years, most likely reflecting improved detection of RR-TB with Xpert; however, access to treatment has remained a challenge. In 2011, prior to Xpert rollout, only 5,643 patients were reported to have started second-line treatment, representing just 56% of the 10,085 patients identified by the National Health Laboratory Service (NHLS) and notified to WHO that year [1]. In 2013, when the rollout of Xpert was largely complete, 10,663 patients were reported to be treated, 72% of the 14,881 notified patients (revised notified figure, personal communication, Nazir Ismael, 2016) [1]. Given the lack of unique patient identifiers, notifications based on laboratory data are likely to include duplicate results from the same patient and may therefore overestimate RR-TB burden. Similarly, the number of treated RR-TB patients in South Africa is derived from a national electronic DR-TB treatment register (EDRWeb), for which data entry is centralised in specialised TB treatment centres in each province, potentially missing patients who receive treatment in decentralised treatment sites. As a result, the treatment gap may be substantially different from that calculated using these routine data sources.

In this study we aimed to determine the proportion of patients with laboratory-diagnosed RR-TB who started second-line treatment and the time to treatment start, comparing cohorts drawn from 2011 (before Xpert availability) and 2013 (after widespread Xpert availability). We further aimed to assess patient-level, geographic, and programmatic factors, such as the decentralisation of RR-TB treatment, associated with second-line treatment initiation.

## Methods

Ethics approval for the study was obtained from the University of Cape Town and the London School of Hygiene & Tropical Medicine. Programmatic approvals were obtained from the South African National Department of Health, provincial departments of health, metropolitan health departments, and relevant health districts and facilities. Approvals were also obtained to access relevant data from three large private laboratories operating in South Africa and the National Department of Correctional Services.

### Study design

The study was a retrospective observational cohort study across all nine South African provinces. Two retrospective cohorts comprised approximately 300 RR-TB patients from each province, sampled sequentially from January 1 onwards in 2011 and 2013. The primary outcome was the proportion of individuals who initiated second-line treatment within 6 mo from the date the NHLS laboratory received the specimen on which the diagnosis of RR-TB was based. Six months was considered as the maximum time to consider treatment initiation within the same diagnostic episode.

### Participants

Patients with RR-TB were included regardless of previous TB treatment history, although in order to include predominantly newly diagnosed RR-TB patients, those with a previous RR-TB diagnosis within the 6 mo prior to the cohort inclusion period were excluded, as were those currently receiving second-line treatment. Inclusion was based on any RR-TB result, regardless of laboratory method. Patients were retrospectively followed up using a range of data sources (see below) to determine and verify the primary outcome of second-line treatment initiation. Data collection was conducted from 13 January 2014 through 24 April 2015, allowing a minimum of 6 mo follow-up time for both cohorts. The study was retrospective so that the conduct of the study would not influence treatment initiation within the 6-mo time period of interest through active follow-up of patients at health facilities.

Time to treatment (days) was determined as the difference between the date the diagnostic specimen (specimen from which RR-TB was diagnosed) was received in the NHLS laboratory and the date second-line treatment was initiated. Specimen receipt in the laboratory was used instead of the specimen collection date due to the unreliability of collection data in this field. A treatment regimen was considered to be second-line if it contained at least two second-line agents, including at least one of a fluoroquinolone or a second-line injectable agent. Patients for whom no previous RR-TB diagnosis could be found were defined as newly diagnosed with RR-TB, while those with prior RR-TB diagnoses (beyond the 6 mo immediately before the inclusion period) were defined as previously diagnosed with RR-TB. Previously diagnosed RR-TB patients were further classified according to whether they had previously received second-line treatment.

### Data sources

Cohorts were established based on routine laboratory data from the NHLS in South Africa. All public-sector TB laboratory testing is done by the NHLS, and test details are captured on a laboratory information system. Only public-sector data were used as these were expected to cover the vast majority of diagnosed cases in South Africa; most patients diagnosed in the private sector are referred to the public sector for treatment and would therefore appear in the public sector records. NHLS data are held in a central repository, and duplicate patients are identified initially by an algorithm that matches according to health care facility medical record numbers. After data extraction of all RR-TB diagnoses in the relevant time periods and 6 mo prior, potential further duplicates were identified using in-house software based on approximate text matching. Potential matches were identified based on first name and surname separately and in combination. Potential matches with exactly matching dates of birth were confirmed automatically, while remaining matches were reviewed manually by at least two researchers, with a third reviewer if the first two reached different conclusions. Overall, 19% of >68,000 potential matches identified were confirmed.

**Fig 1:**
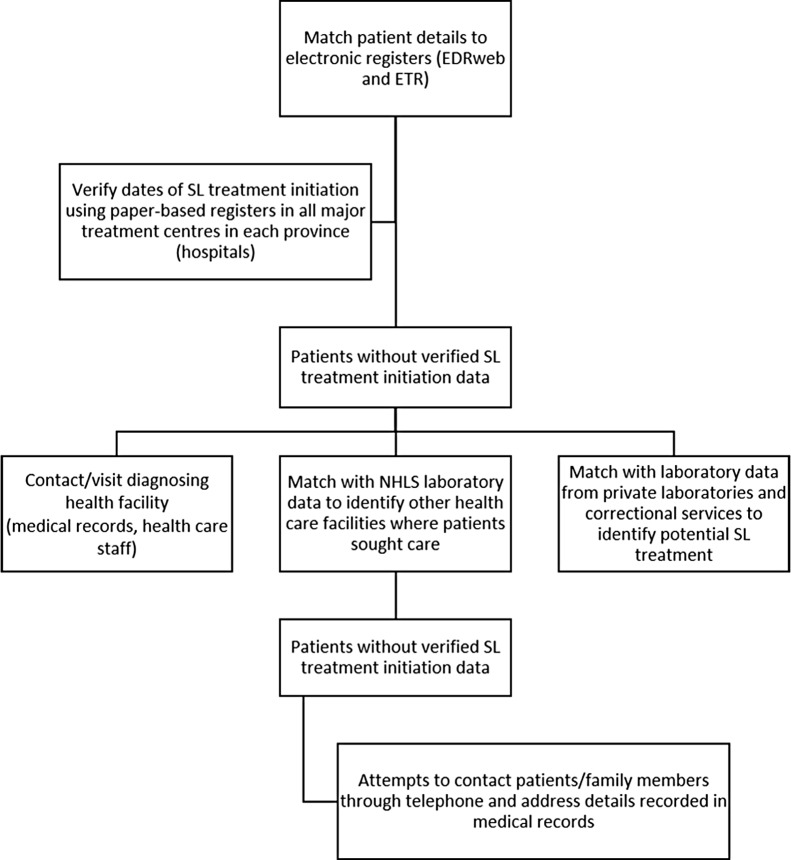
Schematic diagram illustrating the stepwise process for follow-up of cohort patients. EDR, EDR.Net; NHLS, National Health Laboratory Service; SL, second-line.

Determination of second-line treatment initiation followed a stepwise process (Fig 1). First, cohort patients were matched using combinations of names, date of birth, and location to the two main electronic TB treatment registers in South Africa; the DR-TB treatment register (EDRWeb, from the South African National Department of Health, United States President’s Emergency Plan for AIDS Relief, and US Centers for Disease Control and Prevention Global AIDS Program) and the first-line TB treatment register (ETR.Net). Data in these registers are collated centrally by the National Department of Health. All potential matches were verified manually. In addition, all patients, including those already matched to the electronic registers, were also tracked through paper-based treatment registers at all major treatment centres (hospitals and large decentralised treatment sites) in each province. Second, patients without verified second-line treatment start dates were then traced individually, starting from the diagnosing health care facility through referral and treatment facilities, to determine potential treatment initiation. This process included review of individual medical records where available and direct follow-up with health facility staff. Third, remaining patients without second-line treatment start dates were then tracked through all NHLS laboratory records (any laboratory test) nationally to determine care seeking at any other facility in any province. Patient names were also linked to laboratory results in both the private sector (three major private laboratories in South Africa) and from correctional services in each province in order to further determine potential places and dates of second-line treatment initiation. Finally, attempts were made to directly contact remaining patients without second-line treatment initiation data, or their family members, through telephone and address details sourced from medical records in the diagnosing health care facility. Overall, >950 separate health care facilities were visited at least once through the study.

Previous and subsequent NHLS laboratory records were identified for each patient to determine previous RR-TB diagnoses and previous or current second-line treatment. Patients were deemed to have been followed up if any record of the patient beyond the initial laboratory report could be found at a health facility or in laboratory records. Given that lack of follow-up could also represent a single presentation at a health facility, all eligible patients were included in analyses, regardless of follow-up. In addition to data from the diagnostic test and the primary outcome of second-line treatment initiation, other data variables including age, sex, HIV status, second-line treatment facility, and date of death (when available) were collected.

### Sample size determination

Both the burden of RR-TB and population size vary significantly across the nine South African provinces. In addition, given varying strategies for RR-TB management, significant variations in both the percentage of patients who initiate treatment and the time to treatment are expected across provinces. In order to inform policy, valid provincial estimates of the percentage of diagnosed patients initiating second-line treatment and a comparison between 2011 and 2013 for this percentage were required. The sample size for each province was therefore based on an estimated 50% treatment initiation in 2011, with a 15% change in 2013 as the minimum significant difference to detect. The resulting sample size required was 240 per cohort at 90% power. This was increased to 300 to allow for duplicate patients and patients found to be ineligible during follow-up. This resulted in an overall sample size of approximately 5,000, which was considered feasible given the logistics of following up large patient cohorts across a large number of health facilities and through routine data sources.

### Statistical methods

Because sampling in each province did not reflect variation in the burden of RR-TB across the nine provinces, post-stratification sampling weights were applied to derive national-level estimates of the percentage of patients who initiated treatment and median time to treatment. Weights were calculated based on the time needed to reach the final sample size of RR-TB patients in each province as a proxy for the burden of diagnosed RR-TB in each province. An estimate of the overall national burden was calculated by multiplying the final sample size for each province by 1 y/[time taken to enrol the sample from each province]. The population weight for the *i*^th^ province was then calculated as the percentage contribution from province *i* to the national burden divided by the percentage contribution of province *i* to the sample.

Time-to-event analyses were conducted using Cox regression and were not censored for loss to follow-up or mortality. Data on mortality were not available for the majority of patients who did not initiate second-line treatment. Among the patients who did not initiate treatment, a proportion were found through records to have been still “in care” across the 6-mo period of potential treatment initiation. However, the majority were not identified through any records in any health facility for most of the 6-mo period. Differences in proportions were assessed with Pearson chi-squared analyses, while medians were compared using the Mann–Whitney U test. Data were analysed with SPSS Statistics (IBM, version 23). Data were deposited in the Dryad Digital Repository [6].

## Results

### Description of cohorts

**Fig 2:**
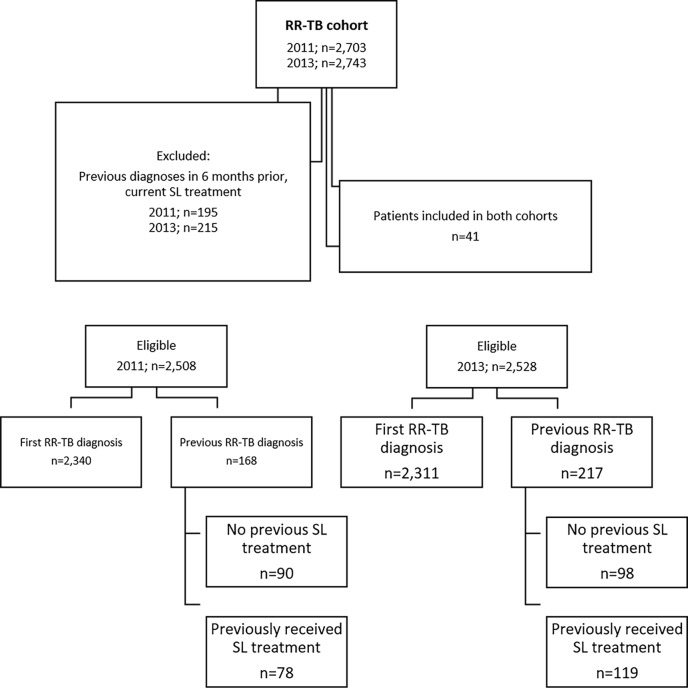
Flow diagram showing initial inclusion, exclusions, and determination of newly and previously diagnosed rifampicin-resistant tuberculosis. RR-TB, rifampicin-resistant tuberculosis; SL, second-line.

The initial 2011 pre-Xpert cohort included 2,703 patients, while 2,743 patients were included in the 2013 post-Xpert cohort. During data collection and verification, 410 (7.5%) patients were excluded as they were found to have had a previous RR-TB diagnosis in the 6 mo prior to the cohort period or were determined to have been receiving second-line treatment at the time of the inclusion RR-TB laboratory result (Fig 2). Of the remaining eligible patients, 98% (2,463 in 2011 and 2,486 in 2013) were defined as followed up through relevant data sources.

The 2011 and 2013 cohorts were similar in terms of age, sex, and HIV status (Table 1). HIV status was known for 82% (2,060/2,508) of patients in the 2011 cohort and 87% (2,189/2,528) in the 2013 cohort, with 63% (1,577) and 66% (1,663) found to be HIV positive, respectively. Among the two cohorts, 93% (2,340) and 91% (2,311) of patients in 2011 and 2013, respectively, were determined through follow-up to be newly diagnosed with RR-TB; the remainder had records of previous diagnoses (not in the previous 6 mo), both with and without record of any prior second-line treatment (Fig 2).

**Table 1:** Demographic and clinical data by cohort.

Characteristic	2011 cohort	2013 cohort
**Total eligible**	2,508	2,528
**Female**	1,181 (47%)	1,192 (47%)
**Age (median, interquartile range)**	35, 27–43	35, 27–43
**Missing age**	14	8
**HIV status**		
Negative	483 (19%)	526 (21%)
Positive	1,577 (63%)	1,663 (66%)
Unknown	448 (18%)	339 (13%)
**Smear status**		
Negative	853 (34%)	577 (23%)
Positive	1,314 (52%)	913 (36%)
Missing	341 (14%)	1,038 (41%)
**Newly diagnosed RR-TB**	2,340 (93%)	2,311 (91%)
**Laboratory test that first detected rifampicin resistance (new RR-TB only)**		
Xpert	88 (4%)	1,368 (59%)
Line probe assay	1,694 (72%)	801 (35%)
Phenotypic DST	558 (24%)	142 (6%)
**Diagnosing facility (new RR-TB only)**		
Primary health care	1,539 (66%)	1,564 (68%)
Secondary hospital	536 (23%)	534 (23%)
Tuberculosis hospital	152 (7%)	106 (5%)
Tertiary hospital	90 (4%)	85 (4%)
Other	23 (1%)	22 (1%)

Data are given as *n* (percent) unless otherwise indicated. Data on diagnostic test that first detected rifampicin resistance and facility where specimen was collected are given for newly diagnosed RR-TB patients only.

DST, drug susceptibility testing; RR-TB, rifampicin-resistant tuberculosis.

Among newly diagnosed RR-TB patients in the 2013 cohort, HIV prevalence ranged from 54% to 75% across the nine provinces (Table 2). The majority of new RR-TB patients in both cohorts and across provinces submitted specimens for diagnosis in primary care facilities, followed by secondary- and tertiary-level hospitals and a smaller percentage from specialised TB hospitals (Table 1).

**Table 2:** New rifampicin-resistant tuberculosis patients and their HIV status, diagnosing facility, and Xpert diagnosis across the nine South African provinces (2013 cohort).

Characteristic	Province								
	1	2	3	4	5	6	7	8	9
***N* (RR-TB diagnoses)**	287	290	283	272	279	261	286	284	286
**New RR-TB diagnosis**	266 (93%)	268 (92%)	264 (93%)	256 (94%)	261 (94%)	231 (89%)	267 (93%)	259 (91%)	239 (84%)
**New RR-TB patients**									
HIV negative	64 (24%)	49 (18%)	19 (7%)	40 (16%)	42 (16%)	36 (16%)	37 (14%)	78 (30%)	95 (40%)
HIV positive	165 (62%)	182 (68%)	186 (71%)	193 (75%)	176 (67%)	165 (71%)	175 (66%)	148 (57%)	128 (54%)
HIV status unknown	37 (14%)	37 (14%)	59 (22%)	23 (9%)	43 (17%)	3 (13%)	55 (21%)	33 (13%)	16 (7%)
Diagnostic specimen from PHC	188 (71%)	206 (77%)	145 (55%)	159 (62%)	157 (60%)	136 (59%)	190 (71%)	206 (80%)	177 (74%)
Xpert diagnosis	158 (59%)	189 (71%)	153 (58%)	145 (57%)	216 (83%)	76 (33%)	164 (61%)	169 (65%)	98 (41%)

Data are given as *n* (percent).

PHC, primary health care; RR-TB, rifampicin-resistant tuberculosis.

### RR-TB diagnosis

The diagnostic test that first detected rifampicin resistance among new RR-TB patients varied between the two cohorts. There was a small percentage of patients in the 2011 cohort diagnosed using Xpert; these were primarily from pilot implementation or research sites. Despite complete rollout of Xpert testing, only 59% (1,368/2,311) of new RR-TB patients from the 2013 cohort were diagnosed initially with Xpert. The remainder were diagnosed primarily with a line probe assay (Hain Lifescience, Germany), either performed directly on a smear specimen or after a positive TB culture (Table 1). To assess if diagnosis with a test other than Xpert was due to non-availability of Xpert, we determined the date that Xpert was first used routinely in each district. Among the 943 patients in the 2013 cohort not diagnosed with Xpert, 42 (5%) were diagnosed from specimens sent to the laboratory before Xpert was used routinely.

Diagnosis with Xpert varied considerably across provinces, ranging from 33% to 83% in 2013 (Table 2). Among newly diagnosed RR-TB patients in the 2013 cohort, the proportion of HIV-positive and HIV-negative patients who were diagnosed initially with Xpert was not different, 60% (916/1,518) and 56% (258/460), respectively (*p* = 0.10).

The median time between the date the NHLS laboratory received the diagnostic specimen and the date the result was reviewed by the laboratory, used as a proxy for laboratory diagnostic time, reduced from 38 d (interquartile range [IQR] 10–67) in 2011 to 2 d (IQR 0–15) in 2013 among new RR-TB patients (*p <* 0.001). In 2013, the laboratory diagnostic time was 1 d (IQR 0–1) for new patients diagnosed with Xpert, 29 d (IQR 13–46) for patients diagnosed with line probe assay (including both direct testing on specimens and after culture), and 47 d (IQR 37–57) for patients diagnosed with phenotypic DST.

### Second-line treatment initiation

**Fig 3:**
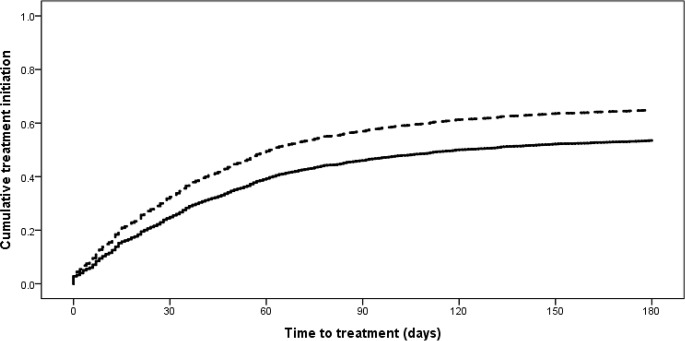
Time to treatment initiation from diagnostic specimen for new rifampicin-resistant tuberculosis patients from the 2011 and 2013 cohorts (*p <* 0.001). Solid line: 2011 cohort; dashed line: 2013 cohort.

**Fig 4:**
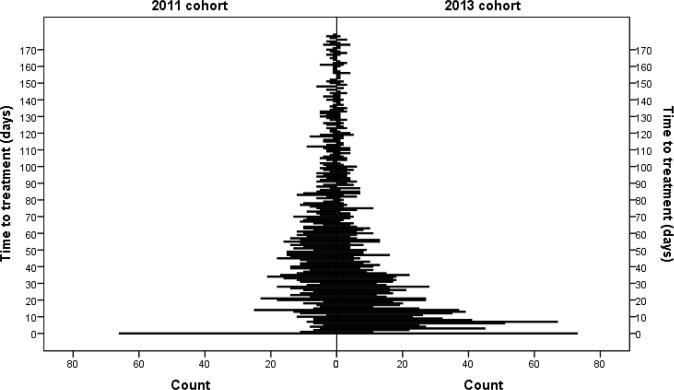
Distribution of time to treatment by cohort.

Second-line treatment initiation within 6 mo of the diagnostic specimen increased from 55% (95% CI 53%–57%) among 2,340 newly diagnosed RR-TB patients in 2011 to 63% (95% CI 61%–65%) of 2,311 patients in 2013 (*p <* 0.001). The median time to treatment among new RR-TB patients starting treatment within 6 mo was reduced by half from 2011 to 2013, decreasing from 44 d (IQR 20–69) to 22 d (IQR 2–43) (*p <* 0.001) (censored at 6 mo). In 2013, only 10% (95% CI 8%–11%) of 2,311 new RR-TB patients had initiated treatment by the national target of 5 d from diagnosis [7]. Fig 3 shows the difference in time to treatment initiation over 6 mo for the 2011 and 2013 cohorts (*p <* 0.001), and Fig 4 shows the distribution of time to treatment for each cohort (data in both figures not weighted for sampling method). Across both cohorts, 4% of patients not diagnosed with Xpert (2,252 in 2011 and 943 in 2013) were reported as starting treatment on the same day as the diagnostic specimen was received in the laboratory. These patients were primarily diagnosed through specialist hospitals, suggesting empiric treatment initiation based on high suspicion of RR-TB. Overall, across both cohorts, 2% of patients who initiated treatment within 6 mo did so in a different province to that in which they were diagnosed.

**Fig 5:**
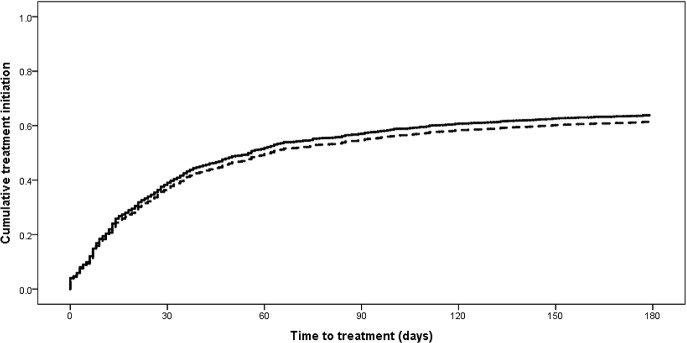
Time to treatment initiation from diagnostic specimen for new rifampicin-resistant tuberculosis patients in the 2013 cohort diagnosed with Xpert compared to other methods (*p <* 0.001). Solid line: diagnosed with Xpert; dashed line: diagnosed by other method.

In the 2013 cohort, there was no difference in the proportion of new RR-TB patients initiating treatment within 6 mo among those diagnosed with Xpert (*n* = 1,368) compared to those diagnosed by other methods (*n* = 943), 64% (95% CI 61%–67%) and 62% (95% CI 59%–65%), respectively (*p* = 0.39). However, the median time to treatment among these patients was reduced: 15 d (IQR 1–29) for patients diagnosed with Xpert compared to 32 d (IQR 7–57) for patients diagnosed by other methods (*p <* 0.001; Fig 5).

There were significant differences in both the proportion of new RR-TB patients initiating treatment and the median time to treatment across the nine South African provinces in 2013 (Table 3). Seven provinces showed significantly increased treatment initiation proportions between 2011 and 2013, while the median time to treatment decreased significantly across all nine provinces (Table 3).

**Table 3:** Percentage of patients who initiated treatment, time to treatment, site of treatment initiation, and recording in national drug-resistant tuberculosis register by cohort and province (new rifampicin-resistant tuberculosis patients).

Province	SL treatment start within 6 mo, *N* (percent, 95% CI)		Median time to SL treatment (days)		Started on SL treatment in PHC, *N* (percent)		Started on SL treatment in tertiary/TB hospital, *N* (percent)		Recorded in national DR-TB register, *N* (percent)	
	2011	2013	2011	2013	2011	2013	2011	2013	2011	2013
1	167 (62%, 56–68)	193 (73%*, 67–78)	34	22*	7 (4%)	13 (7%)	158 (95%)	164 (85%*)	117 (70%)	139 (72%)
2	128 (48%, 42–54)	176 (66%*, 58–74)	65	18*	72 (56%)	87 (49%)	5 (4%)	10 (6%)	68 (53%)	91 (52%)
3	135 (51%, 45–57)	134 (51%, 45–56)	48	26*	13 (10%)	14 (10%)	110 (82%)	88 (66%*)	61 (45%)	36 (27%*)
4	107 (48%, 41–54)	158 (62%*, 58–65)	68	21*	9 (8%)	10 (6%)	59 (55%)	104 (66%)	74 (69%)	78 (49%*)
5	121 (46%, 39–52)	144 (55%*, 46–65)	47	33*	1 (1%)	6 (4%)	117 (97%)	128 (89%*)	44 (36%)	30 (21%*)
6	107 (39%, 33–45)	129 (56%*, 49–63)	65	41*	6 (6%)	6 (5%)	89 (83%)	113 (88%)	48 (45%)	50 (39%)
7	106 (37%, 31–42)	160 (60%*, 52–68)	46	18*	6 (6%)	12 (8%)	1 (1%)	1 (1%)	54 (51%)	72 (45%)
8	131 (54%, 47–60)	179 (69%*, 58–80)	40	20*	16 (12%)	27 (15%)	53 (41%)	49 (27%*)	95 (73%)	111 (64%)
9	180 (74%, 69–80)	175 (73%, 68–78)	36	13*	109 (61%)	115 (66%)	58 (32%)	47 (27%)	132 (73%)	134 (77%)
Total**	1,182 (55%, 53–57)	1,448 (63%*, 61–65)	44	22*	239 (23%)	290 (19%*)	650 (62%)	704 (56%*)	693 (62%)	745 (53%*)

*p < 0.05 compared to 2011.

**Weighted for sampling method (note that the reported percentages may not match the percentages obtained by dividing the raw numbers due to weighting to account for the sampling method).

DR-TB, drug-resistant tuberculosis; PHC, primary health care; SL, second-line; TB, tuberculosis.

In 2011, 62% the new RR-TB patients started second-line treatment (*n* = 1,182) at tertiary level or specialist TB hospitals. This proportion dropped significantly in 2013 to 56% (*n* = 1,448) but remained the most common type of facility for initiating second-line treatment (Table 3). Primary health care facilities were responsible for treatment initiation in 23% and 19% of patients in 2011 and 2013, respectively, but this proportion varied significantly across provinces, ranging from 4% to 66% in 2013 (Table 3). Among new RR-TB patients found to have initiated second-line treatment, only 62% and 53% in 2011 and 2013, respectively, were recorded in the national electronic DR-TB treatment register. This proportion decreased significantly between 2011 and 2013 in three provinces and was <50% in five provinces in 2013 (Table 3).

### Previously diagnosed rifampicin-resistant tuberculosis patients

Overall, 8% (385/5,036) of patients, from both cohorts, were found to have a prior diagnosis of RR-TB. Among those for whom no previous second-line treatment could be identified, the time between the first recorded RR-TB diagnosis and the diagnostic result for which they were included in the cohort was a median of 19 mo (Table 4). Treatment initiation (for the current episode) was higher among these patients compared to those with records of prior treatment, 76% and 9%, respectively, with no significant difference in median time to treatment (Table 4).

**Table 4:** For previously diagnosed rifampicin-resistant tuberculosis patients, time from first recorded rifampicin-resistant tuberculosis diagnosis, HIV status, treatment initiation, and time to treatment by previous second-line treatment status (not weighted for sampling method).

Characteristic	Previously diagnosed—no previous SL treatment identified, *N* = 188	Previously diagnosed—previous SL treatment identified, *N* = 197
**Median time from first recorded RR-TB diagnosis (months)**	19 (IQR 9–29)	Not able to be determined
**Smear status**		
Negative	53 (28%)	62 (32%)
Positive	108 (57%)	66 (34%)
Missing	27 (14%)	69 (65%)
**HIV status**		
Negative	67 (36%)	58 (29%)
Positive	115 (61%)	133 (68%)
Unknown	6 (3%)	6 (3%)
**SL treatment start within 6 mo**	148 (76%)	19 (9%)
**Median time to SL treatment (days)**	35 (IQR 6–66)	28 (IQR 0–65)

IQR, interquartile range; RR-TB, rifampicin-resistant tuberculosis; SL, second-line.

### Univariate analysis of factors associated with second-line treatment initiation

The percentage of new RR-TB patients initiating second-line treatment was similar across age groups, with the exception of patients aged 55 y and older (56%, 95% CI 49%–63%), who had a lower median treatment initiation rate than to patients aged 45–54 y (71%, 95% CI 66%–76%) (*p <* 0.001) (Table 5). The median time to treatment was longer for children aged 0–15 y (41 d, IQR 5–77) compared to individuals aged 16–24 y (21 d, IQR 2–41) (*p* = 0.04); children were predominantly treated in hospitals and not in primary care. There was no difference in the percentage of patients who initiated treatment or time to treatment by sex.

**Table 5:** Second-line treatment initiation within 6 mo, time to treatment, and site of treatment initiation by age, sex, HIV status, diagnostic facility, and diagnostic test (new rifampicin-resistant tuberculosis patients, 2013 cohort).

Characteristic	*N*	Started SL treatment, *N* (percent*, 95% CI)	Median time to SL treatment, days* (IQR)	Started SL treatment in PHC, *N* (percent*, 95% CI)
**Age (years)**				
0–15	64	42 (74%, 63–86)	41 (5–77)	1 (2%, 0–6)
16–24	281	191 (66%, 60–71)	21 (2–41)	45 (24%, 18–30)
25–34	734	461 (63%, 60–66)	20 (1–39)	96 (20%, 17–24)
35–44	672	400 (59%, 55–62)	21 (1–42)	79 (17%, 13–21)
45–54	346	243 (71%, 66–76)	28 (9–48)	49 (19%, 14–24)
55+	204	110 (56%, 49–63)	20 (1–39)	20 (16%, 9–24)
**Sex**				
Female	1,092	675 (61%, 58–63)	23 (1–45)	142 (19%, 16–22)
Male	1,217	773 (65%, 62–67)	21 (4–41)	148 (19%, 16–21)
**HIV status**				
Negative	460	382 (83%, 79–86)	22 (2–42)	7 (24%, 20–29)
Positive	1,517	1,039 (67%, 65–70)	21 (1–42)	88 (17%, 14–19)
Missing	332	27 (9%, 6–12)	36 (0–77)	195 (30%, 11–47)
**HIV positive**				
Diagnosed with Xpert	916	601 (67%, 64–70)	24 (2–28)	123 (18%, 15–21)
Diagnosed by other method	601	452 (67%, 63–71)	34 (10–59)	72 (15%, 11–18)
**Diagnostic facility type**				
PHC	1,563	1,013 (63%, 61–66)	22 (2–42)	255 (25%, 22–28)
Secondary hospital	533	283 (56%, 52–60)	24 (0–48)	28 (8%, 5–11)
Tuberculosis hospital	106	88 (82%, 75–88)	6 (0–25)	2 (2%, 1–4)
Tertiary hospital	85	48 (58%, 49–69)	25 (13–38)	4 (8%, 0–15)
Other	22	16 (77%, 54–99)	20 (2–37)	1 (8%, 1–29)

All percentages and times are weighted for sampling method.

*All percentages and times are weighted (note that the reported percentages may not match the percentages obtained by dividing the raw numbers due to weighting to account for the sampling method).

IQR, interquartile range; PHC, primary health care; SL, second-line.

Treatment initiation among HIV-positive patients (67%, 95% CI 65%–70%) was lower than among HIV-negative patients (83%, 95% CI 79%–86%) (*p <* 0.001) (Table 5), while there was a low level of treatment initiation among patients with unknown HIV status (9%, 95% CI 6%–12%). There was no difference in treatment initiation between HIV-positive patients diagnosed with Xpert (67%, 95% CI 64%–70%) compared to those diagnosed with other tests (67%, 95% CI 63%–71%); however, the median time to treatment was significantly reduced with Xpert diagnosis (24 d, IQR 2–28, versus 34 d, IQR 10–59) (*p <* 0.001) (Table 5). Not unexpectedly, the highest treatment initiation rate was seen among patients diagnosed directly through TB hospitals (82%, 95% CI 75%–88%). Treatment initiation was lower among patients diagnosed in either secondary (56%, 95% CI 52%–60%) or tertiary (58%, 95% CI 49%–69%) hospitals compared to those diagnosed in primary health care facilities (63%, 95% CI 61%–66%) (*p <* 0.001) (Table 5).

## Discussion

The proportion of diagnosed RR-TB patients initiating second-line treatment in South Africa improved significantly between 2011 and 2013, increasing from 55% to 63%. However, the remaining patients, 37% in 2013, represent the treatment gap and are likely to contribute to ongoing transmission in the community and to suffer from high mortality. This estimate is higher than reports of initial loss to follow-up among all TB cases in different settings in South Africa, which range from 11% to 25% in recent studies [8–10], suggesting that there are additional barriers to initiation of second-line treatment for DR-TB. These barriers might be expected to include high pre-treatment mortality, given long delays to receiving drug susceptibility test results, and more restricted access to second-line treatment, resulting most commonly from the need for referral for treatment initiation.

Routine estimates of the treatment gap are derived from aggregate data from different data sources: diagnosed patients from laboratory reports and treated patients from the national DR-TB register. The treatment gap of 37% in our 2013 cohort is similar to the 38% of cases not started on treatment in that year according to the routine reporting system. However, this similarity may be coincidental; in the current study only 53% of patients in 2013 who started second-line treatment were actually recorded in the national DR-TB treatment register in South Africa. The remaining 47% were not registered in the treatment register and yet were initiated on second-line treatment within 6 mo of the diagnostic specimen. This suggests that the estimate of treatment initiation percentage using routine data is flawed. Potentially, this flawed calculation could be due to inaccurate algorithms for matching duplicate laboratory results and to long delays in registering patients in the national register, so that registrations reflect patients diagnosed in previous years. In addition, it could be due to multiple treatment episodes for individual patients being counted as separate patients in the register. This disconnect between laboratory-reported notifications and patients initiating treatment points to the need for an integrated diagnosis and treatment register for programme monitoring.

The low registration rate of treated patients in the national register is concerning in itself, and likely reflects programmatic issues in the management of the database. In many provinces, patients can be registered only by staff at centralised facilities, suggesting that patients started on treatment at lower health system levels may not be registered in the system. This situation may also have contributed to the drop in the percentage registered between 2011 and 2013. It is also possible that registration of patient data in the system is severely delayed in many settings, also contributing to the difference between the cohorts. In order to provide effective and timely feedback, such a registration system should ideally include all diagnosed cases, not just those treated, and be updated regularly at all treatment initiation sites.

Between 2011 and 2013, South Africa implemented the Xpert MTB/RIF test for all individuals being investigated for TB. Universal access to DST is recommended by the World Health Organization and was expected to substantially increase case detection, given that the majority of RR-TB cases are among previously untreated TB patients, who were not offered DST under previous guidelines. Indeed, in South Africa, the annual number of RR-TB cases notified to WHO increased by more than 80% between 2011 and 2014 [1,11]. In South Africa, the implementation of Xpert is likely to have contributed substantially to the drop in median time to treatment between 2011 and 2013. Although there is little empiric evidence, the month prior to starting TB treatment is likely to represent the time period with the highest level of infectiousness, and, as such, for RR-TB, a gain of close to a month between presentation to a health facility and starting second-line treatment is likely to contribute to reducing ongoing transmission [5].

However, diagnosis using the Xpert test did not significantly impact the treatment gap. In 2013, by 6 mo, similar proportions of patients diagnosed with Xpert had initiated second-line treatment compared to those diagnosed by other methods. These data suggest that the reduction in the treatment gap between 2011 and 2013 is likely to be due to other programmatic improvements. In 2011, the South African National Department of Health released a new policy supporting decentralisation and deinstitutionalisation of DR-TB care [12]. The 2011 policy allowed for ambulatory treatment of DR-TB through primary care services, and was driven primarily by a shortage of specialist TB hospital beds and the presence of waiting lists for admission and therefore treatment initiation [12]. While the extent of decentralised treatment provision varies across South African provinces, several decentralised RR-TB programmes in South Africa have demonstrated treatment gaps of less than 10% [13,14], suggesting that decentralised care can contribute to reducing the treatment gap. While data from the current study show some improvement in the proportion of RR-TB patients initiating treatment at lower levels of the health system, 49% still started treatment at tertiary or specialised TB hospitals in 2013, and there was no change in the proportion starting treatment in primary care settings. However, there was substantial variation across South African provinces reflecting variable implementation of the decentralisation policy [15]. Variations in how the national decentralisation policy has been interpreted and implemented across provinces, along with other potential improvements in programme management, such as improved communication of results and referral systems, may have confounded the impact of Xpert on time to treatment between 2011 and 2013.

Additionally, the median time to treatment, even among patients diagnosed with Xpert, was still substantially higher than the 5-d national target [7], although this target may not be realistic given the need for counselling and pre-treatment testing. Given that the laboratory diagnosis was made in a median of 1 d for patients diagnosed with Xpert, most of this delay is due to other health system factors, likely to include delays in communicating laboratory results to health care facilities, difficulties in recalling patients, and the need for referral and/or hospital admission where required. The delays to treatment initiation shown here confirm that Xpert implementation is insufficient in itself, and needs to be combined with improvements in accessibility of treatment and delivery of patient-centred services, as demonstrated in some settings [14,16].

While the majority of South African provinces showed improvement in the proportion of patients initiating treatment within 6 mo of diagnosis between 2011 and 2013, treatment initiation within 6 mo ranged from 53% to 74% in 2013, suggesting large treatment gaps even in the best performing provinces. Improvements in the median time to treatment were more consistent across provinces; however, treatment delay still ranged between 15 and 36 d across provinces in 2013. Overall, only two provinces showed significant levels of treatment initiation in primary care.

HIV-positive patients were less likely than HIV-negative patients to initiate treatment. Given that the median time to treatment was similar by HIV status, and there was no difference in the proportion diagnosed initially with Xpert, this difference is likely due to higher mortality among HIV-positive patients. Rapid mortality, preventing appropriate treatment initiation, was shown earlier in some settings in South Africa [17]. These data highlight the importance of both earlier presentation for testing and further reductions in treatment delay among HIV-positive RR-TB patients. Particularly long delays in treatment initiation were observed among the relatively small proportion of children aged <15 y with RR-TB (<3%). These children were almost entirely started on treatment in hospital settings, most likely contributing to the treatment delay. Given the paucibacillary nature of TB disease in children, these patients likely represent a small fraction of the total community RR-TB burden in children [18]. Improving case detection among this vulnerable group will likely necessitate greater responsibility for management in primary care settings.

Although Xpert has lower sensitivity than culture for TB diagnosis [3], the proportion of RR-TB patients initially diagnosed with Xpert was surprisingly low at 59%. Given that only a small proportion of patients were diagnosed in districts without Xpert availability, the range in this proportion across the provinces may be indicative of varied practices in implementation of Xpert. It may be that Xpert is not being utilised for all presumptive TB patients in some settings, whereas in others, further diagnostic testing of patients with negative Xpert tests may be inadequate.

Across both the 2011 and 2013 cohorts, approximately 8% of patients were found to have had previous RR-TB diagnoses. This was despite efforts to preferentially include newly diagnosed patients by excluding those with previous diagnoses in the 6 mo prior to the diagnostic specimen and all of those already receiving second-line TB treatment. Given the large treatment gap, this relatively high proportion is not surprising and represents a group of patients who are reappearing in the health system without adequate treatment. The long duration of time from the first RR-TB diagnosis that could be located suggests that these patients may well have chronic disease and are likely to contribute significantly to ongoing transmission in the community.

In this study, we were unable to assess the reasons for non-initiation of second-line treatment, although they likely include early mortality, clinician failure to identify laboratory results showing RR-TB, inability to locate patients, and lack of options for patients to receive treatment close to where they reside. Unfortunately, mortality data were inconsistently available, particularly among patients who did not initiate second-line treatment. Few of these patients had national identification numbers available with which to verify mortality status through the national deaths registry in South Africa. Additionally, attempts to directly contact patients and/or family members were largely unsuccessful, due to changed or incorrect telephone numbers and insufficiently detailed addresses. As a result, we may have underestimated treatment initiation in circumstances where patients moved long distances to seek treatment and where names and dates of birth were recorded incorrectly. However, given the high level of follow-up among cohort patients, this contribution is likely to be low. Time to treatment may also have been underestimated as we did not include the time for specimen transport in our estimate. Finally, it is possible that, due to the longer period between diagnosis and follow-up for the 2011 cohort compared to the 2013 cohort, more patients who initiated treatment may have been missed in the 2011 cohort compared to the 2013 cohort.

South Africa has demonstrated global leadership in implementing universal access to DST through access to Xpert for all presumptive TB patients, contributing to substantially increased case detection for RR-TB. While a significant treatment gap remained in 2013, given increased case detection, a larger proportion received second-line treatment, with reduced delays to treatment initiation. The treatment gap in South Africa is in contrast to global figures, where the vast majority of the global burden of RR-TB remains undiagnosed [1]. However, the large burden of diagnosed, but untreated, RR-TB patients should and is acting as a catalyst to drive strategies to provide effective treatment to all diagnosed patients. The National Department of Health is currently rolling out plans to appoint one “linkage officer” per district with the aim of enhancing patient tracing and linkage to appropriate treatment for RR-TB patients [19]. Further health system strengthening will likely be required to fully exploit the benefit of new technologies for diagnosis of TB and drug resistance.

## Supporting information

S1 Text STROBE checklist for cohort studies. (DOC)

S2 Text Prospective analysis plan. (DOCX)

## Abbreviations

DR-TB: drug-resistant tuberculosis
DST: drug susceptibility testing
IQR: interquartile range
NHLS: National Health Laboratory Service
RR-TB: rifampicin-resistant tuberculosis
TB: tuberculosis
WHO: World Health Organization

## References

[1] World Health Organization. Global tuberculosis report 2015. WHO/HTM/TB/2015.22. Geneva: World Health Organization; 2015.

[2] Dowdy DW, Chaisson RE, Maartens G, Corbett EL, Dorman SE. Impact of enhanced tuberculosis diagnosis in South Africa: a mathematical model of expanded culture and drug susceptibility testing. Proc Natl Acad Sci U S A. 2008;105(32):11293–8. 10.1073/pnas.0800965105 18695217 PMC2516234

[3] Boehme CC, Nicol MP, Nabeta P, Michael JS, Gotuzzo E, Tahirli R, et al Feasibility, diagnostic accuracy, and effectiveness of decentralised use of the Xpert MTB/RIF test for diagnosis of tuberculosis and multidrug resistance: a multicentre implementation study. Lancet. 2011;377(9776):1495–505. 10.1016/S0140-6736(11)60438-8 21507477 PMC3085933

[4] Harris RC, Grandjean L, Martin LJ, Miller AJ, Nkang JE, Allen V, et al The effect of early versus late treatment initiation after diagnosis on the outcomes of patients treated for multidrug-resistant tuberculosis: a systematic review. BMC Infect Dis. 2016;16(1):193. 27142682 10.1186/s12879-016-1524-0 PMC4855810

[5] Menzies NA, Cohen T, Lin HH, Murray M, Salomon JA. Population health impact and cost-effectiveness of tuberculosis diagnosis with Xpert MTB/RIF: a dynamic simulation and economic evaluation. PLoS Med. 2012;9(11):e1001347 10.1371/journal.pmed.1001347 23185139 PMC3502465

[6] Cox H, Dickson-Hall L, Ndjeka N, van’t Hoog A, Grant AD, Cobelens F, et al. Data from: Delays and loss to follow-up before treatment of drug-resistant tuberculosis following implementation of Xpert MTB/RIF in South Africa: a retrospective cohort study. Dryad Digital Repository. 10.1371/journal.pmed.1002238 PMC5319645 28222095

[7] South African National AIDS Council. National strategic plan (NSP) for HIV and AIDS, TB and STIs 2012–2016. Pretoria: South African National AIDS Council; 2013.

[8] Cox H, Mbhele S, Mohess N, Whitelaw A, Muller O, Zemanay W, et al Impact of Xpert MTB/RIF implementation for TB diagnosis in a high TB and HIV prevalence primary care clinic in South Africa: a pragmatic randomised trial. PLoS Med. 2014;11(11):e1001760 10.1371/journal.pmed.1001760 25423041 PMC4244039

[9] Churchyard GJ, Stevens WS, Mametja LD, McCarthy KM, Chihota V, Nicol MP, et al Xpert MTB/RIF versus sputum microscopy as the initial diagnostic test for tuberculosis: a cluster-randomised trial embedded in South African roll-out of Xpert MTB/RIF. Lancet Glob Health. 2015;3(8):e450–7. 10.1016/S2214-109X(15)00100-X 26187490

[10] Van Den Handel T, Hampton KH, Sanne I, Stevens W, Crous R, Van Rie A. The impact of Xpert((R)) MTB/RIF in sparsely populated rural settings. Int J Tuberc Lung Dis. 2015;19(4):392–8. 10.5588/ijtld.14.0653 25859993

[11] World Health Organization. Global tuberculosis control: WHO report 2012. Geneva: World Health Organization; 2012.

[12] South African National Department of Health. Management of drug-resistant tuberculosis: policy guidelines. Pretoria: South African National Department of Health; 2011.

[13] Naidoo P, du Toit E, Dunbar R, Lombard C, Caldwell J, Detjen A, et al A comparison of multidrug-resistant tuberculosis treatment commencement times in MDRTBPlus line probe assay and Xpert(R) MTB/RIF-based algorithms in a routine operational setting in Cape Town. PLoS ONE. 2014;9(7):e103328 10.1371/journal.pone.0103328 25079599 PMC4117508

[14] Cox H, Daniels J, Muller O, Nicol M, Cox V, Van Cutsem G, et al Impact of decentralized care and the Xpert MTB/RIF test on rifampicin-resistant tuberculosis treatment initiation in Khayelitsha, South Africa. Open Forum Infect Dis. 2015;2(1):ofv014 10.1093/ofid/ofv014 26034764 PMC4438894

[15] South African National Department of Health, World Health Organization. Towards universal health coverage: report of the evaluation of South Africa drug resistant TB programme and its implementation of the policy framework on decentralised and deinstitutionalised management of multidrug resistant TB. Pretoria: South African National Department of Health; 2016.

[16] Loveday M, Wallengren K, Voce A, Margot B, Reddy T, Master I, et al Comparing early treatment outcomes of MDR-TB in decentralised and centralised settings in KwaZulu-Natal, South Africa. Int J Tuberc Lung Dis. 2012;16(2):209–15. 10.5588/ijtld.11.0401 22236922 PMC3281510

[17] Gandhi NR, Andrews JR, Brust JC, Montreuil R, Weissman D, Heo M, et al Risk factors for mortality among MDR- and XDR-TB patients in a high HIV prevalence setting. Int J Tuberc Lung Dis. 2012;16(1):90–7. 10.5588/ijtld.11.0153 22236852 PMC3302205

[18] Dodd PJ, Sismanidis C, Seddon JA. Global burden of drug-resistant tuberculosis in children: a mathematical modelling study. Lancet Infect Dis. 2016;16(10):1193–201. 10.1016/S1473-3099(16)30132-3 27342768

[19] Dreyer A, Omar S, Nanoo A, Koornhof H, Ismail N. Public health action to reduce the burden of rifampicin resistant tuberculosis. Communicable Dis Surveill Bull. 2015;13(2):47–51. Johannesburg: National Institute for Infectious Diseases.

