# Supplementary material for: Delays and loss to follow-up before treatment of drug-resistant tuberculosis following implementation of Xpert MTB/RIF in South Africa: A retrospective cohort study
Source: PLoS Med. 2017 Feb 21;14(2):e1002238. doi: 10.1371/journal.pmed.1002238 (PMC5319645; doi:10.1371/journal.pmed.1002238)
Supplement: S1 Text — (DOC) [file pmed.1002238.s001.doc]

STROBE Statement—Checklist of items that should be included in reports of ***cohort studies***

**Paper title: Delays to treatment and the treatment gap for drug-resistant tuberculosis in South Africa: a national retrospective cohort study assessing the impact of Xpert implementation**

|  | Item No | Recommendation | Response |
| --- | --- | --- | --- |
| **Title and abstract** | 1 | (*a*) Indicate the study’s design with a commonly used term in the title or the abstract | The title includes the study design: “retrospective cohort study” |
|  |  | (*b*) Provide in the abstract an informative and balanced summary of what was done and what was found | The abstract describes the study design, methods and results, lines 23-62. |
| Introduction | | |  |
| Background/rationale | 2 | Explain the scientific background and rationale for the investigation being reported | The background and rationale are described in paragraph 3 of the introduction, lines 68-82. |
| Objectives | 3 | State specific objectives, including any prespecified hypotheses | The specific objective is stated in paragraph 4 of the introduction, lines 132-136. There were no pre-specified hypotheses. |
| Methods | | |  |
| Study design | 4 | Present key elements of study design early in the paper | The study design is described in paragraph 2 of the methods, and describes the formation of two cohorts, the sampling strategy and sample size. |
| Setting | 5 | Describe the setting, locations, and relevant dates, including periods of recruitment, exposure, follow-up, and data collection | The setting is described in line 145; “…cohort study across all nine South African provinces”.  The start dates for cohort inclusion are given in lines 146-147; “…sampled sequentially from January 1st onwards in 2011 and 2013 respectively”.  Duration of follow-up is described in line 147-150; “The primary outcome was the proportion of individuals who initiated second-line treatment within six months from the date of the specimen on which the diagnosis of RR-TB was based.”  Dates of data collection are given in lines 157-158; “Data collection was conducted from January 2014 through April 2015”. |
| Participants | 6 | (*a*) Give the eligibility criteria, and the sources and methods of selection of participants. Describe methods of follow-up | Inclusion criteria are described under ‘participants’, lines 152-155.  Data sources for inclusion of participants are described under ‘data sources’, lines 172-184.  Data sources and methods for the determination of the primary outcomes are also described under ‘data sources’, lines 185-204, and in figure 1. |
|  |  | (*b*) For matched studies, give matching criteria and number of exposed and unexposed | Not applicable |
| Variables | 7 | Clearly define all outcomes, exposures, predictors, potential confounders, and effect modifiers. Give diagnostic criteria, if applicable | The primary outcome of second-line treatment initiation is defined in lines 164-166; “A treatment regimen was considered to be second-line if it contained at least two second-line agents, including at least one of a fluoroquinolone or a second-line injectable agent”.  Other definitions are given in lines 161-170 and include definitions of time to treatment and criteria for defining newly diagnosed RR-TB cases. |
| Data sources/ measurement | 8* | For each variable of interest, give sources of data and details of methods of assessment (measurement). Describe comparability of assessment methods if there is more than one group | Data sources and methods of data attainment are described under ‘data sources’ in the methods. |
| Bias | 9 | Describe any efforts to address potential sources of bias | As the study aimed to estimate a valid provincial estimates of second-line treatment initiation, similar sample sizes were sought in each province despite disproportionate RR-TB burdens. To address this sampling bias in determining national estimates, sampling weights were calculated based on the time taken to reach the required sample size in each province, and used to adjust the primary outcome of treatment initiation and the time to treatment initiation. This is described in the ‘statistical methods’ section, lines 226-235. |
| Study size | 10 | Explain how the study size was arrived at | The sample size took into account both the need for provincial estimates of the primary outcome and logistical issues around collecting data across all nine provinces in South Africa. This is described in lines 213-224. |
| Quantitative variables | 11 | Explain how quantitative variables were handled in the analyses. If applicable, describe which groupings were chosen and why | Most variables were categorical, with the exception of time to treatment, which was described using medians and interquartile ranges, given non-normal distribution. Age was grouped into 10-year categories, with the exception of children aged 0-15 as this is the South African definition of children. |
| Statistical methods | 12 | (*a*) Describe all statistical methods, including those used to control for confounding | Statistical methods are described in lines 226-243, and include the description of weighting, as above. |
|  |  | (*b*) Describe any methods used to examine subgroups and interactions | Only univariate analyses were conducted. Sub-analyses were conducted for HIV positive patients in the study and those diagnosed with the Xpert test. |
|  |  | (*c*) Explain how missing data were addressed | All missing data is described in table 1. Categories of missing data were included for missing HIV status and missing smear status. |
|  |  | (*d*) If applicable, explain how loss to follow-up was addressed | Data were not censored for death or loss to follow up in the ‘time to event’ analyses, as this data was inconsistently available. This is stated in lines 236-241. |
|  |  | (*e*) Describe any sensitivity analyses | Not applicable |
| Results | | |  |
| Participants | 13* | (a) Report numbers of individuals at each stage of study—eg numbers potentially eligible, examined for eligibility, confirmed eligible, included in the study, completing follow-up, and analysed | Numbers of study participants are described in lines 246-251 and in figure 2. |
|  |  | (b) Give reasons for non-participation at each stage | Reasons for non-participation are given in figure 2. |
|  |  | (c) Consider use of a flow diagram | Figure 2 |
| Descriptive data | 14* | (a) Give characteristics of study participants (eg demographic, clinical, social) and information on exposures and potential confounders | The study participants are described in table 1. |
|  |  | (b) Indicate number of participants with missing data for each variable of interest | See table 1 |
|  |  | (c) Summarise follow-up time (eg, average and total amount) | The primary outcome was treatment initiation within 6 months; all participants had at least 6 months follow-up. Hence follow-up beyond 6 months is not described. |
| Outcome data | 15* | Report numbers of outcome events or summary measures over time | The primary outcome is described in lines 293-321 and in table 3 by province. Events over time are shown in figures 3 and 5. |
| Main results | 16 | (*a*) Give unadjusted estimates and, if applicable, confounder-adjusted estimates and their precision (eg, 95% confidence interval). Make clear which confounders were adjusted for and why they were included | The primary outcome of treatment initiation across provinces is given in table 3 and national estimates for South Africa, weighted to account for the sampling strategy is given in lines 294-296. All estimates are given with 95% confidence intervals. |
|  |  | (*b*) Report category boundaries when continuous variables were categorized | Not applicable |
|  |  | (*c*) If relevant, consider translating estimates of relative risk into absolute risk for a meaningful time period | Not applicable |
| Other analyses | 17 | Report other analyses done—eg analyses of subgroups and interactions, and sensitivity analyses | Univariate analysis of factors associated with treatment initiation is given in table 5. |
| Discussion | | |  |
| Key results | 18 | Summarise key results with reference to study objectives | See lines 376-384 in discussion. |
| Limitations | 19 | Discuss limitations of the study, taking into account sources of potential bias or imprecision. Discuss both direction and magnitude of any potential bias | Limitations are discussed in lines 476-490. |
| Interpretation | 20 | Give a cautious overall interpretation of results considering objectives, limitations, multiplicity of analyses, results from similar studies, and other relevant evidence | See discussion |
| Generalisability | 21 | Discuss the generalisability (external validity) of the study results | Not applicable. |
| Other information | | |  |
| Funding | 22 | Give the source of funding and the role of the funders for the present study and, if applicable, for the original study on which the present article is based | The source of funding is given in line 143, methods; “This study was funded by the Bill and Melinda Gates Foundation.” |

*Give information separately for exposed and unexposed groups.
