# Supplementary material for: Delays and loss to follow-up before treatment of drug-resistant tuberculosis following implementation of Xpert MTB/RIF in South Africa: A retrospective cohort study
Source: PLoS Med. 2017 Feb 21;14(2):e1002238. doi: 10.1371/journal.pmed.1002238 (PMC5319645; doi:10.1371/journal.pmed.1002238)
Supplement: S2 Text — (DOCX) [file pmed.1002238.s002.docx]

**Supplementary information – Prospective analysis plan**

**Title: Linkage to Care for Drug Resistant TB Patients Following Xpert Implementation in South Africa**

## Primary aim: To assess linkage to care and critical determinants of ‘non linkage’ and delay among people with a rifampicin resistant (RR) laboratory result before and after Xpert implementation.

## Secondary aims: To determine the positive predictive value of a RR Xpert result for South Africa overall, and to describe the extent of potentially inappropriate treatment of patients who have discordant RR results.

**Methods**

In order to meet the study aims, the project will include two phases.

Phase 1 will be a retrospective systematic consecutive sample of patients in South Africa with a RR result will be conducted starting on the 1st January 2011 and 2013, and continuing until the sample size of 300 patients is achieved for each of the 9 provinces, utilising data from the NHLS Central Data Warehouse. Following the preliminary analysis of the datasets obtained through phase 1, phase 2 of the protocol will use this data to inform a series of descriptive, qualitative sub-studies to assess patient level and health system factors contributing to expedited treatment initiation or failure to initiate second-line treatment.

# Specific objectives of phase 1

## Primary objectives

- Describe the proportion of people with an RR result who start MDR treatment in each province and nationally.
- Describe the proportion of patients who have commenced MDR treatment within 1, 2, 3 and 6 months of diagnosis for patients with a RR Xpert result and patients with RR TB diagnosed through other tests.
- Identify factors associated with failure to start MDR treatment in patients with RR TB diagnosed through any test.
- To compare patients diagnosed with RR TB before and after Xpert roll out with regard to:
- The time between specimen arrival at the laboratory and initiation of second-line TB treatment
- The number, proportion and characteristics of patients who start second-line TB treatment within 1, 2,3 and 6 months after specimen collection
- The number of patients not starting treatment after 6 months following specimen collection.
- Mortality that occurred within 6 months after specimen collection

## Secondary objectives

- Describe the proportion of patients with a RR Xpert result for whom confirmatory test results are available for rifampicin susceptibility.
- Estimate the positive predictive value of a RR Xpert result against the reference standard of LPA and DST.
- Describe the proportion of patients placed on MDR treatment following discordant Xpert and other DST results.

## Analysis plan

For the cohort analyses based on time to treatment (KM curves and Cox regression), the time interval starts at the date the specimen (for TB diagnosis) was received at NHLS. This date will be available for all patients. The date of specimen collection would be more appropriate, but is likely not available for all patients. The date of sample arrival at NHLS will be used as a proxy, and data of patients for whom both dates are available will be used to assess possible distortion of the results. The time interval ends at the date second-line treatment (the event of interest) is initiated. Patients who did not start second-line treatment and died within 6 months will be censored at the date of death. Patients who started first-line treatment or did not start second-line treatment and did not die will be censored at 6 months.

**Variations to the analysis plan**

Our initial analysis plan outlined calculating the proportion of patients who initiate treatment within 1, 2, 3 and 6 months of diagnosis. During analysis, it was decided to present this more clearly as a time to event analysis.

While, we have presented some data on factors that may influence time to treatment initiation, we felt that a full multi-level analysis was beyond the scope of the initial paper. More detailed analysis of factors within each province forms part of a subsequent analysis that is currently underway.

Our analysis plan included objectives related to the mortality among RR-TB not starting treatment. Unfortunately, this data was not able to be collected for a large proportion of patients, and hence this was not done.

As part of the primary aim to assess determinants of non-linkage and delay, a qualitative study was undertaken in parallel to the quantitative cohort study (phase 2 of the study). This data is still under analysis and will be published separately.

Similarly, the secondary objective of determining the positive predictive value (PPV) for Xpert MTB/RIF is part of a sub-study assessing PPV and the nature of discordance in rifampicin-resistance testing, that is also currently under analysis. This analysis will form a separate research article.
